# Numerical mesoscale tissue model of electrochemotherapy in liver based on histological findings

**DOI:** 10.1038/s41598-022-10426-2

**Published:** 2022-04-20

**Authors:** Helena Cindric, Gorana Gasljevic, Ibrahim Edhemovic, Erik Brecelj, Jan Zmuc, Maja Cemazar, Alenka Seliskar, Damijan Miklavcic, Bor Kos

**Affiliations:** 1 Faculty of Electrical Engineering, University of Ljubljana, Trzaska cesta 25, 1000 Ljubljana, Slovenia; 2 Institute of Oncology Ljubljana, Zaloska cesta 2, 1000 Ljubljana, Slovenia; 3 Faculty of Medicine, University of Ljubljana, Vrazov trg 2, 1000 Ljubljana, Slovenia; 4 Faculty of Health Sciences, University of Primorska, Polje 42, 6310 Izola, Slovenia; 5 University of Ljubljana, Veterinary Faculty, Gerbiceva ulica 60, 1000 Ljubljana, Slovenia

**Keywords:** Biomedical engineering, Computational science, Liver cancer, Computational models

## Abstract

Electrochemotherapy (ECT) and irreversible electroporation (IRE) are being investigated for treatment of hepatic tumours. The liver is a highly heterogeneous organ, permeated with a network of macro- and microvasculature, biliary tracts and connective tissue. The success of ECT and IRE depends on sufficient electric field established in whole target tissue; therefore, tissue heterogeneity may affect the treatment outcome. In this study, we investigate electroporation in the liver using a numerical mesoscale tissue model. We numerically reconstructed four ECT experiments in healthy porcine liver and computed the electric field distribution using our treatment planning framework. We compared the computed results with histopathological changes identified on microscopic images after treatment. The mean electric field threshold that best fitted the zone of coagulation necrosis was 1225 V/cm, while the mean threshold that best fitted the zone of partially damaged liver parenchyma attributed to IRE was 805 V/cm. We evaluated how the liver macro- and microstructures affect the electric field distribution. Our results show that the liver microstructure does not significantly affect the electric field distribution on the level needed for treatment planning. However, major hepatic vessels and portal spaces significantly affect the electric field distribution, and should be considered when planning treatments.

## Introduction

Electrochemotherapy (ECT) is a localized tumour treatment that combines the use of chemotherapeutic agents with the application of short high-voltage electric pulses to tissue. The application of pulses causes a transient increase in cell membrane permeability—reversible electroporation—that facilitates the transport of ions and molecules to which the membrane is otherwise impermeable or poorly permeable. Reversible electroporation significantly enhances the local cytotoxic effect of agents with intracellular targets, such as bleomycin and cisplatin^1–4^. ECT is already an established treatment for cutaneous and subcutaneous tumours (e.g. skin malignancies, head and neck tumours), due to its high effectiveness, relatively simple application and good cosmetic results^5,6^. Based on its effectiveness for superficial lesions, ECT is now also being investigated for treatment of various deep-seated tumours^2,3,7,8^. A significant part of recent studies is focused on treatment in the liver; results indicate that ECT is a feasible and effective treatment option for primary^9–11^ and secondary liver tumours^12–15^.

A prerequisite for a successful electroporation-based treatment is complete coverage of target tissue volume with sufficiently high electric field. For this purpose, numerical models of various scales are being developed for accurate prediction of electroporation in target tissue; from bulk tissue models used for treatment planning^16–19^, to models of densely packed cells^20–23^, models of single cells, and models of cell membrane electroporation^24,25^. The liver is a highly heterogeneous organ, permeated with a network of blood vessels and biliary tracts. Several studies have already shown the importance of considering liver macrostructures (large blood vessels and bile ducts), when constructing models for electroporation-based treatments in the liver^26–28^. Moreover, liver parenchyma has a distinct microstructure consisting of functional units called hepatic lobules, each containing a centrilobular vein (CV). Hepatic lobules relate to a network of connective tissue, blood vessels and bile ducts (portal triads), called the interlobular septa. For modelling purposes, the lobules are usually represented as prisms with hexagonal cross sections of 1000–2000 μm diameter with CVs in the center (80–187 μm diameter), and separated by gaps (~ 50 μm) representing the interlobular septa^29,30^. The electric field distribution depends on the electrical properties of the medium. This is especially important when treating target volumes that contain tissues with significantly different conductivities, as the majority of voltage drop, and consequently electric field strength, occurs in tissues with low conductivity^18,27,31,32^. The natural heterogeneity of the liver structure may have an impact on the electric field distribution and consequently on the outcome of electroporation-based treatments in liver.

In this study, we investigate ECT in the liver using a mesoscale tissue model that is comparable to findings from microscopic images after treatment. In a recent translational animal model study^33^, we examined whether ECT with bleomycin causes clinically significant damage to normal liver tissue with respect to large blood vessels and bile ducts. In our present study, we numerically reconstructed four of the experiments performed in that study^33^, and computed the electric field distribution in tissue with our previously developed treatment planning framework^19,34–36^. The aim of this study was to compare the computed electric field distribution with the histopathological changes observed in tissue after treatment, and to evaluate how the liver structures affect the electric field distribution. We determined the electric field thresholds that best correspond to the changes present in tissue after ECT, and evaluated the temperature increase and probability of thermal damage to tissue, with special focus on sensitive anatomical structures such as vessels and bile ducts. We investigated whether the liver microstructure (i.e. hepatic lobules, septa and CVs) and the variability in its’ electrical conductivity affect the electric field distribution in any extent relevant for comparison with histopathological findings.

## Results

### Electric field thresholds and tissue heating

**Figure 1:**
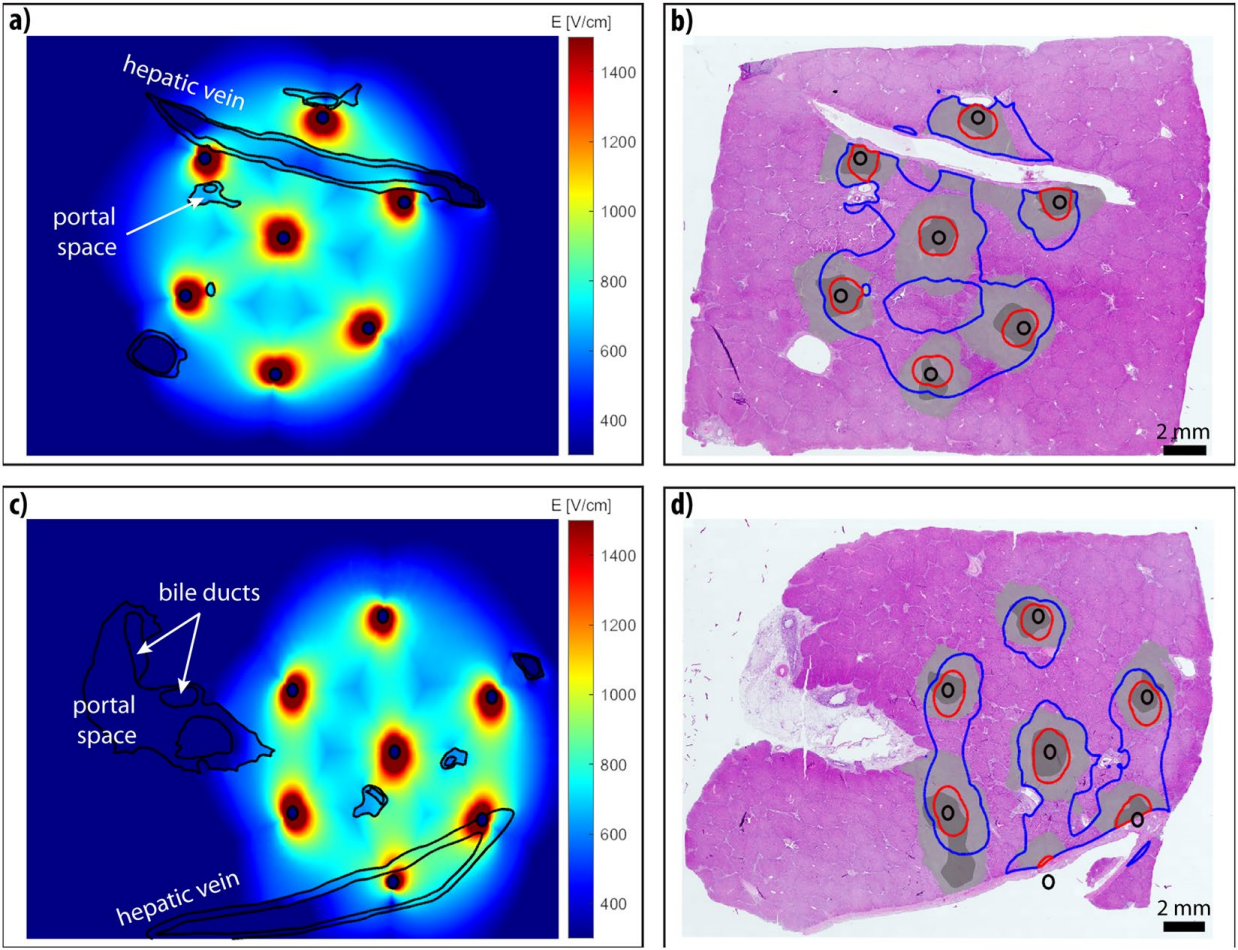
Reconstruction results of cases S1 (**a**, **b**) and S2 (**c**, **d**) with hexagonal geometry electrodes. (**a**, **c**) Computed electric field distribution. Geometric entities are outlined in black. (**b**, **d**) Segmented Zone A (dark grey) and Zone B (light gray), and computed Zone A (red contour) and Zone B (blue contour) shown as an overlay on the microscopic image of the treated area.

**Figure 2:**
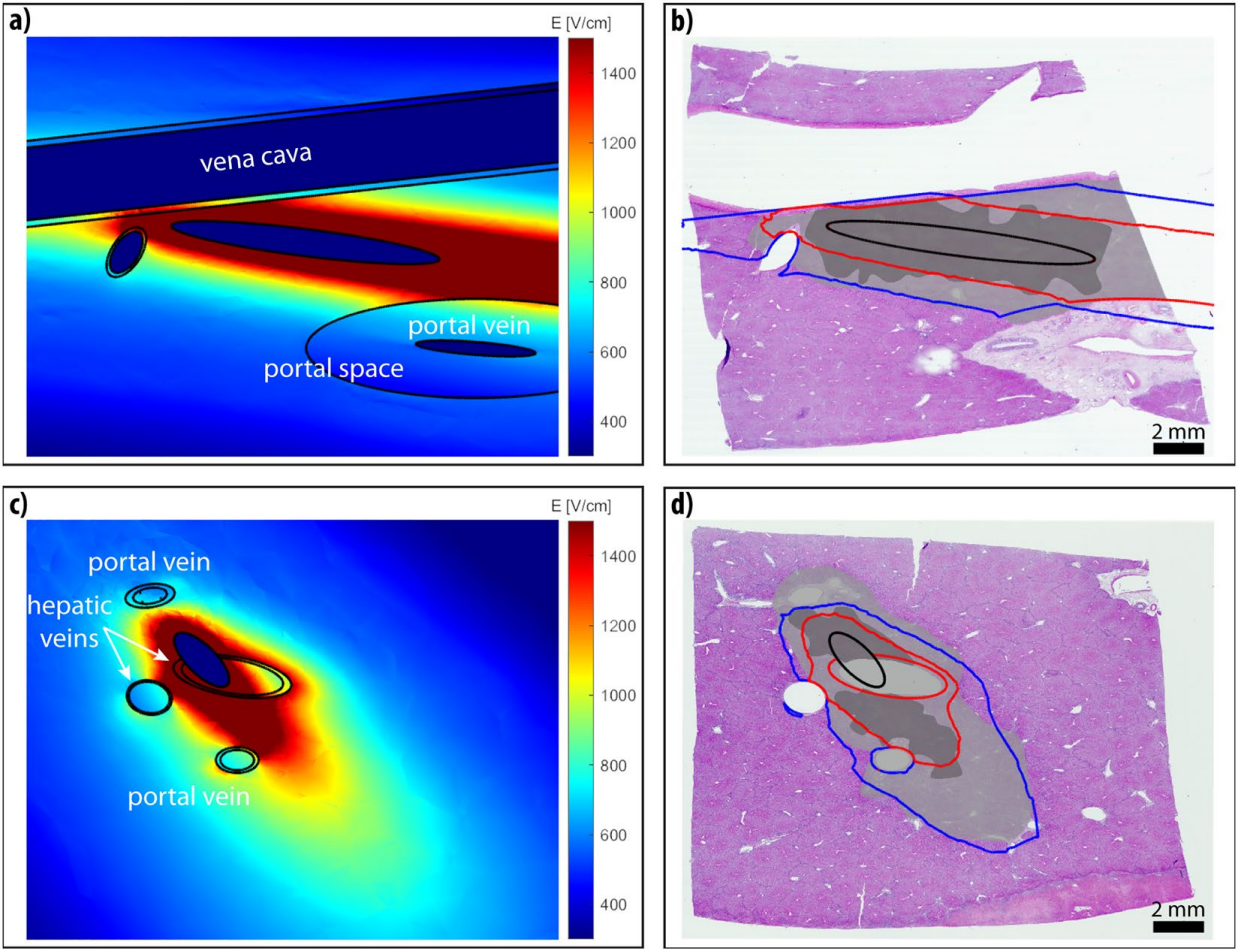
Reconstruction results of cases S3 (**a**, **b**) and S4 (**c**, **d**) with linear geometry electrodes. (**a**, **c**) Computed electric field distribution in a slice of the 3D model, corresponding to the microscopic image. Geometric entities are outlined in black. (**b**, **d**) Segmented Zone A (dark grey) and Zone B (light gray), and computed Zone A (red contour) and Zone B (blue contour) shown as an overlay on the microscopic image of the treated area.

We reconstructed four experiments of liver ECT from the study reported previously^33^ and computed the electric field distribution and heating in treated tissue. We fitted the computed electric field to the microscopic images and determined the thresholds that best fit the two zones of histopathological changes observed in the samples—Zone A, immediately surrounding the electrode insertion site that exhibits coagulation necrosis with complete loss of liver microstructure, and Zone B of partially damaged liver parenchyma. The electric field thresholds corresponding to the two zones for each reconstructed case are presented in Table 1 along with their respective Sørensen-Dice similarity coefficients. The mean thresholds were 1225 ± 52 V/cm for Zone A and 805 ± 59 V/cm for Zone B. The threshold for Zone A encapsulates the potential temperature-related changes and electro-chemical changes at the treated site, while threshold for Zone B is attributed mainly to damage caused by irreversible electroporation (IRE). Figures 1, 2 show the computed electric field distribution and the determined zones for hexagonal and linear geometry electrodes respectively.

**Table 1:** Computed electric field threshold with the best Sørensen-Dice similarity coefficient (DSC) for Zone A (mechanical damage and coagulation necrosis) and Zone B (damage caused by IRE).

Sample/model	Electrode geometry	Threshold [V/cm]		DSC [/]	
		Zone A	Zone B	Zone A	Zone B
S1	Hexagonal	1230	760	0.71	0.74
S2	Hexagonal	1150	800	0.65	0.70
S3	Linear	1260	770	0.67	0.85
S4	Linear	1260	890	0.75	0.87

There was no significant heating observed in tissue. With linear geometry electrodes, the temperature did not exceed 41 °C anywhere in the tissue, while in case of hexagonal electrodes the temperature reached 47 °C only at the electrode surface. Probability of thermal damage was determined by integrating the Arrhenius equation over the time of treatment and was < 1% everywhere in tissue in all four cases; therefore, the tissue necrosis observed in Zone A is not considered to be a direct consequence of elevated temperature in tissue. Nevertheless, a map of maximum temperature distribution for all four cases is shown in Supplementary Fig. S3.

### Liver microstructure and variability in electric properties

We evaluated how the liver microstructure—hepatic lobules, septa and centrilobular veins (CV)—affects the electric field distribution. We compared the electric field distribution computed with numerical models incorporating a varying degree of geometrical detail of the liver parenchyma: a heterogeneous model (hepatic lobules, septa and CVs), a semi-homogenous model (homogeneous hepatic tissue with CVs) and a fully homogenous model (only hepatic tissue). Detailed description of the models is provided in the Methods sub-section “Liver microstructure and parametric study of tissue properties”.

**Figure 3:**
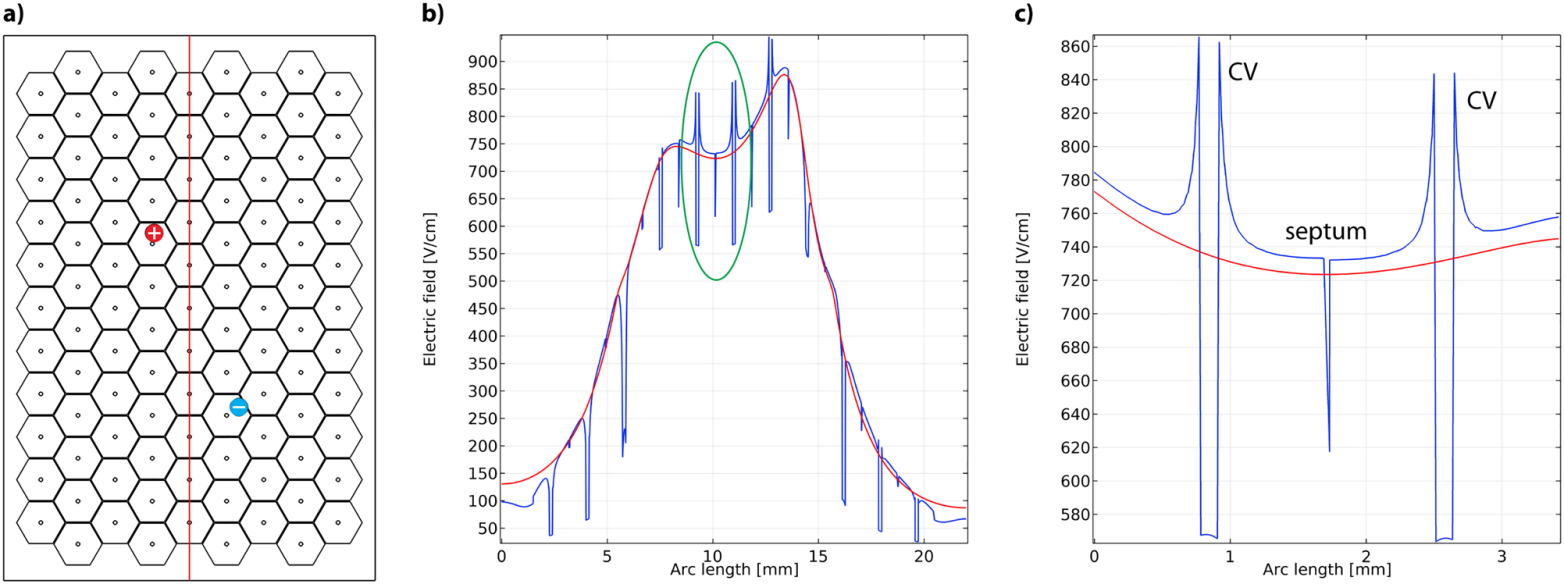
(**a**) A section of the model, showing two electrodes (red–cathode, blue–anode) and the cut-line (red vertical line). **(b)** Electric field strength along the cut-line crossing several septa and centrilobular veins (CV). (**c**) A close up of a section (indicated in green on panel (**b**) showing two lobules with CVs separated by a septum. Red lines on panels (**b**) and (**c**) show the electric field computed with a completely homogenous model.

Figure 3 shows an example of the electric field, computed with the heterogeneous and homogeneous models. In this example, the septa had the same conductivity function shape as the vessel wall (see Supplementary Tables S1 and S2). When crossing the septum domain we observe a drop in electric field strength, however, it resumes its previous value immediately after leaving the septum (Fig. 3b,c). When crossing the domain of the CV we observe a spike at the edge of the CV lumen, which is a consequence of a much higher conductivity of blood within the lumen compared to surrounding tissue, and then a drop in electric field strength in the CV lumen respectively (Fig. 3b,c). This effect was also reported in previous studies on smaller vessels and capillaries^37^. In this example the mean relative error between electric field computed with a heterogeneous model and a fully homogenous model is 7% with a standard deviation of 12%, while the median relative error is 3%. The mean relative error between the electric field computed with a semi-homogenous and a fully homogenous model is 3% with a standard deviation of 10%, while the median relative error is 0.5%.

Using the heterogeneous model, we performed a parametric study, to evaluate how the differences in conductivity of the septa affect the electric field distribution and determined thresholds for Zones A and B. Electric field was computed for all combinations of parameter values of the septa conductivity function (see Methods Section "Liver microstructure and parametric study of tissue properties"), resulting in 72 computations. The electric field thresholds for Zones A and B were calculated for each combination and results were compared to thresholds determined with the semi homogeneous and fully homogeneous models. Row 1 of Table 2 shows the median and mean electric field thresholds, range and standard deviation for both zones, obtained in the parametric study with the heterogeneous model. Rows 2 and 3 of Table 2 show the electric field thresholds for both zones, obtained with the semi-homogeneous and homogeneous models, respectively. We can see that the threshold for Zone B is not affected by any of the parameters studied. If the base conductivity of the septa is much lower than the conductivity of the lobules (e.g., 10% of its base value), the threshold for Zone A is lower than in the homogenous models (660 ± 22 V/cm vs. 850 V/cm respectively). If the base conductivity of the septa is equal or higher than the conductivity of lobules, the threshold for Zone A does not significantly differ from the thresholds from the homogenous models (853 ± 8 V/cm vs. 850 V/cm respectively). The size and location of the transition zone do not affect the threshold. Although the exact conductivity of the interlobular septa is not known, we hypothesize that their conductivity is higher than the conductivity of the lobules because the septa consist of connective tissues, blood vessels and bile ducts. Therefore, we can assume the septa does not affect the electric field thresholds at the mesoscale.

**Table 2:** Electric field thresholds for Zones A and B determined with models with different level of geometric detail. Only one section of the model, containing one active electrode pair, was computed due to computational complexity.

Model	Zone A threshold [V/cm]		Zone B threshold [V/cm]	
	median (range) [V/cm]	mean ± std [V/cm]	median (range) [V/cm]	mean ± std [V/cm]
Heterogeneous (parametric study)	840 (640–860)	810 ± 70	480 (480–490)	480 ± 2
Semi-homogeneous	850	/	480	/
Homogeneous	850	/	470	/

## Discussion

In this study, we compare the electric field distribution, computed in a mesoscale tissue model, with the histopathological changes observed in healthy hepatic tissue after ECT. We numerically reconstructed four ECT procedures, performed in a recent in vivo animal study by Zmuc et al.^33^ and examined how the liver macro- and microstructures affect the electric field distribution. We also investigated the tissue temperature increase due to Joule heating and its’ potential effect on the nearby vessels and bile ducts.

The four studied samples are different from the ones presented in our previous study; special attention was given to select samples where several major hepatic vessels were involved in the treatment area. In three cases at least one electrode was inserted directly into the vessel, while in the fourth case (sample S1) the electrodes were positioned around one of the hepatic veins. The presence of these hepatic vessels affects the gross electric field distribution. In Figs. 1–2, we can see an increase in electric field strength at the side of the vessel perpendicular to the electrodes, and a decrease in field strength at the side parallel to the electrode. This effect is most notable around the lower portal vein in Fig. 2c. Our findings are in agreement with previous studies^26–28^, indicating that presence of larger vessels should not be overlooked, when computing the electric field for electroporation-based treatments. In samples S1, S2 and S4 larger portal spaces containing bile ducts were present in vicinity of the treated zone, however, no damage was observed in the histological examination of the samples. The portal space constitutes mainly of supportive connective tissue (type I collagen fibers, lymphatics, cholangioles) with a higher base electrical conductivity than the surrounding liver parenchyma (0.26 S/m compared with 0.091 S/m^19,27,38^) and is therefore exposed to a lower electric field strength than its surroundings..

No significant tissue heating was observed in our models. This comes at no surprise, since only a small number of pulses is used in ECT. The highest computed temperature reached 47 °C in sample S1 with hexagonal electrodes, but only immediately at the surface of the electrode that was positioned in the hepatic vein. In case of the linear geometry electrodes, the temperature did not exceed 41 °C, since a significantly lower number of pulses is cumulatively applied and a slower repetition rate is used. Probability of thermal damage according to Arrhenius kinetics equation was < 1% everywhere in tissue for all four cases.

In our previous study^33^, we investigated whether ECT with bleomycin causes clinically significant damage to normal liver tissue. Upon histological examination of explanted liver samples, acute changes with clear zonation were observed in the tissue. The area immediately surrounding the electrode insertion site exhibited coagulation necrosis with complete loss of liver microstructure (Zone A; Zones 1–2 in the paper by Zmuc et al.). Surrounding this area was a zone of partially damaged liver parenchyma, which was attributed to irreversible electroporation (IRE) of tissue (Zone B; Zone 3 in the paper by Zmuc et al.). No histological changes were observed in areas exposed to reversible electroporation. Furthermore, the addition of bleomycin (electrochemotherapy) did not cause any difference compared with samples only treated with electric pulses. In our current study, we fitted the computed electric field to the microscopic images of the treated area to determine the threshold values that best fit the appearance of the Zones A and B.

The mean electric field threshold that best fits the appearance of Zone A is 1225 V/cm. Although histological examination of Zone A revealed changes characteristic of coagulation necrosis, in Zmuc et al. we postulated that it is not likely these changes were caused by tissue heating during pulse delivery, which was now also confirmed by our computations. In recent years the role of pH changes in cell death mechanisms is being investigated in electroporation-based treatments^39–44^. The pH change is attributed to the ion transport, which results in a strong acidification at the site of the anode and alcalinization at the site of the cathode, which result in necrotic regions near the electrode insertion site. The necrosis in Zone A observed in our samples may be related to the pH changes around the electrodes, however, further research is needed to verify this speculation. The appearance of Zone B is most likely caused by IRE of tissue. Even though lower pulse amplitudes and pulse number are used in ECT, an area of irreversibly electroporated tissue is also present around the electrodes. Our computations show the best fit with Zone B is achieved with electric field strength of 810 V/cm, which is sufficient to cause IRE of hepatic tissue with reversible electroporation pulse protocols^16,27,45^.

In Zmuc et al.^33^, the estimated thresholds for Zone A were 1500 V/cm for linear and 1200 V/cm for hexagonal electrodes. The electroporated volume and electric field threshold depend on the number and duration of applied electric pulses (i. e. exposure time). With one pair of linear electrodes significantly less pulses are cumulatively delivered to tissue compared to hexagonal electrodes (8 pulses vs. 96 pulses respectively), therefore, a higher threshold for linear geometry electrodes is expected^24^. In present study, however, we found no significant difference between the thresholds for linear and hexagonal electrodes for both zones. We achieved good agreement with the original study for the hexagonal electrodes, however, for linear electrodes our threshold was approximately 300 V/cm lower. The discrepancy between the results could be due to sample selection bias. Although the data was obtained from the same experiments, the thresholds in the present study were determined on samples located at the sites of major hepatic vessels and bile ducts, whereas in the previous study^33^ the thresholds were determined on samples located exclusively in the liver parenchyma and obtained from a different animal. Biological variability and different methods of threshold determination are among the main reasons for the different threshold values reported in literature^16,19,45–48^. Lastly, in Zmuc et al., the thresholds were estimated by matching the radius of Zone A to the distance obtained from the image of field distribution. For electric fields of such high intensity (> 800 V/cm), the transition zone is very short and even an error of 1 mm in the estimated zone radius can result in a difference of 150 V/cm in the electric field threshold. However, in the present study, we used a refined image-based fitting method for threshold determination with a step of 10 V/cm.

In the second part of the study, we investigated whether the microscopic structure of the liver (i.e. hepatic lobules, septa, CVs) and the differences in electrical conductivity of these microstructures affect the electric field distribution during ECT on a level relevant for comparison with microscopic images. The result of our study show, that the inclusion of the liver microstructures does not significantly affect the computed electric field. There is a localized drop in field strength observed in the domain of the septa and CVs (Fig. 3), however, these domains are composed mainly of connective tissue and microvasculature and do not contain cells targeted by electroporation-based treatments, therefore the treatment success should not be negatively affected. When compared to a fully homogenous tissue model (with electrical properties of hepatic lobules, see Table S1 in supplementary materials) there is no significant difference in computed electric field strength. According to the results of our parametric study, the only scenario, where the inclusion of the septa would significantly affect the gross electric field distribution, is if the conductivity of the septa is much lower than the conductivity of surrounding hepatic tissue (e.g. 10%; 0.0091 S/m compared to 0.091 S/m for the septa and lobules, respectively). However, the interlobular septa consist of connective tissue, venules, arterioles and bile ducts, which suggests the conductivity of the septa is in fact higher than the surrounding tissue. Our findings confirm there is no significant difference in gross electric field distribution and the determined thresholds for Zones A and B regardless of the inclusion of the septa and CVs in the model.

In the previous study^33^, Zmuc et al. observed that the damage in Zone B was not distributed equally, as it was more pronounced in the centri- and midlobular areas. This observation might be explained by taking into consideration the vascularization of the hepatic lobules. At the center of each lobule there is a centrilobular vein surrounded by sinusoids (capillaries), while the “wall” of the lobule consists of the vascularized septa and portal tracts. Blood flows through the lobule walls, traverses the sinusoids, and flows into the centrilobular vein^29^. In Fig. 3, we can see pronounced spikes in electric field strength at the lumen of the CVs, which could cause damage to the vessel. This is also in agreement with the histological findings, as the CVs were no longer visible in the damaged areas of Zone B. In the domains of the septa however, the electric field is lower compared to surrounding tissue, which could spare the microvasculature. It is possible that the damage in the centrilobular domain is due to disruption of central vasculature of the lobules, while the outer parts of the lobules are less affected due to still functional vasculature. When evaluating the electric field distribution at a mesoscale, these local fluctuations in electric field strength were not significant.

Our study was mainly limited by its retrospective nature. While in case of hexagonal electrodes, the positions of the electrodes were easily discernible in the histological samples, the soft liver tissue was somewhat deformed during electrode insertion, therefore the inter-electrode distances in the samples were no longer 7.3 mm in all locations within the samples. Already a 1–2 mm difference in inter-electrode distance resulted in significant difference in the determined electric field thresholds. We applied scaling and shearing deformations to the 2D models, however, we were unable to completely eliminate the deformation, therefore small differences in inter-electrode distance remained. On the other hand, in case of linear geometry electrodes, we were able to fix the inter-electrode distance in the model to 2 cm. However, only one electrode site was visible in the sample and the position of the counter electrode had to be determined to the best of our ability, based on the shape of the Zones A and B.

In conclusion, we confirmed that the liver microstructure (hepatic lobules, interlobular septa and centrilobular veins) does not significantly affect the electric field distribution at a mesoscale. The use of a fully homogeneous model of the liver parenchyma is suitable for the numerical computations of electric field in the liver organ, needed for planning electroporation-based treatments. However, major hepatic vessels and portal spaces should be included in the model, as these macrostructures significantly affect the electric field distribution, as already suggested before^27^.

## Methods

### Animal experiments

The animal experiments were performed in the scope of a recent in vivo animal model study by Zmuc et al.^33^, which is reported in accordance with ARRIVE guidelines for reporting of research involving animals. All experiments were performed in accordance with the relevant guidelines and regulations. Regulatory approval for this study was obtained from the National Ethics Committee at The Administration of the Republic of Slovenia for Food Safety, Veterinary, and Plant Protection (U34401-1/2017/4; approval date: 17.03.2017). Experiments were performed on healthy pig liver; six pigs were treated with ECT with bleomycin and two pigs received pulses only, serving as control. The pulses were delivered with the Cliniporator pulse generator (IGEA, Italy) using either fixed hexagonal geometry electrodes or two linear geometry electrodes with 2 cm spacing. The following ECT protocols were used: for linear geometry electrodes 8 × 100 μs, 2000 V electric pulses were delivered with a 1/s repetition rate; for fixed hexagonal geometry electrodes 96 × 100 μs, 730 V electric pulses were delivered with a 5000/s repetition rate. The applied voltage in both electrode geometries corresponded to a 1000 V/cm voltage-to-distance ratio. Two days after the procedure the liver was explanted, cut and fixed in formaldehyde. After 24 h, the specimens were cut into 2–3 μm thick samples, stained with H&E and microscopically examined and photographed. Further details regarding the experiments, treatment protocols and histological analysis are described in^33^.

### Numerical reconstructions

**Figure 4:**
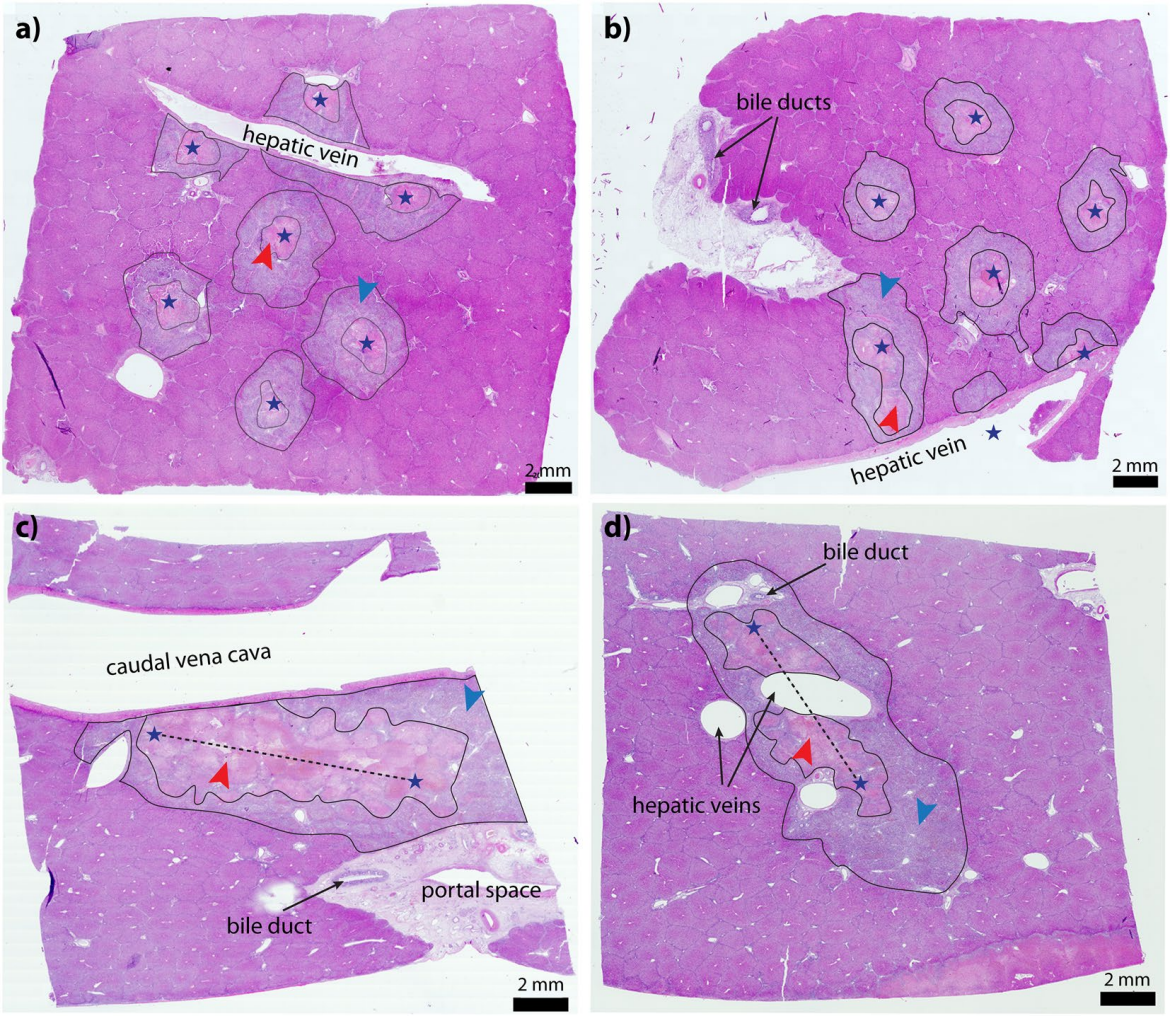
Microscopic images of treated liver 2 days post ECT. Electrode insertion sites are denoted with stars and dashed lines. Portal spaces and major hepatic vessels are contained in the treatment zone. Zones A (red arrowhead) and B (blue arrowhead) are outlined in black. (**a**, **b**) Samples S1 and S2 of animal 1, treated with hexagonal geometry electrodes. (**c**, **d**) Samples S3 and S4 of animal 2, treated with linear geometry electrodes. Since the second electrode was 2 cm away, it is always far from the field of view of these histological sections, so they are not visible.

**Figure 5:**
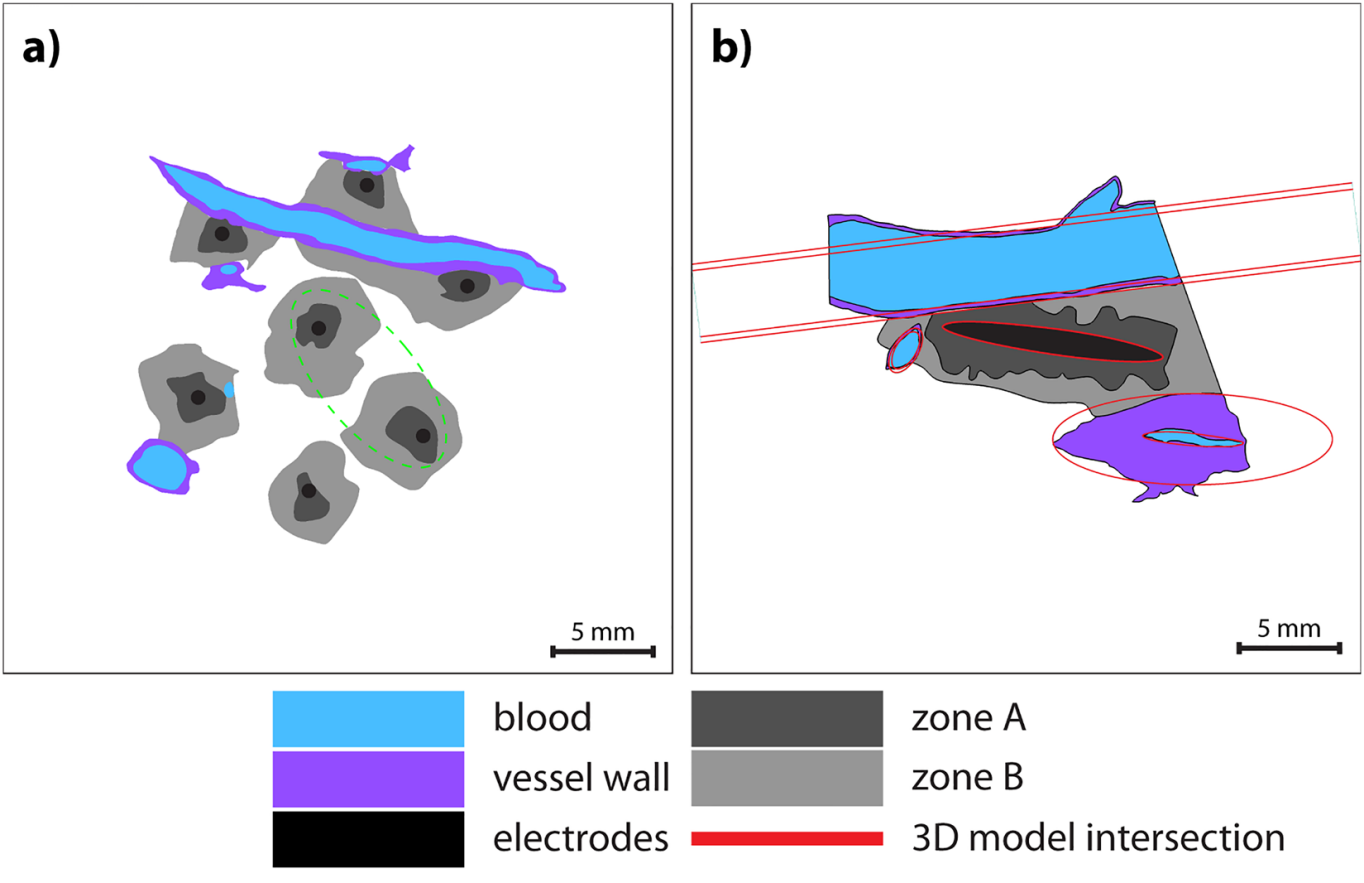
Numerical model geometries. (**a**) 2D model geometry of sample S1 is constructed directly from the outlined microscopic image. Green circle indicates the section of the model, used in the parametric study of septa electrical conductivity. (**b**) A slice from the simplified 3D model with linear electrodes. The location of the slice corresponds to the microscopic image of sample S3. Intersections of the geometric entities with the outlined microscopic image are shown in red.

For our present study, four histological samples from two animals treated with ECT with bleomycin were selected for numerical reconstruction and analysis. The samples were selected from procedures performed at the sites of the major hepatic vessels and portal spaces, and therefore differ from the samples in the work of Zmuc et al. Fig. 4 shows the microscopic images of the selected samples 2 days post treatment. Animal 1 (Fig. 4a,b) was treated with hexagonal geometry electrodes (pig #2 from Zmuc et al.). In sample S1, electrodes were inserted in the liver parenchyma abutting the hepatic vein (a) and in sample S2, two electrodes were inserted into the hepatic vein (b). Animal 2 (Fig. 4c,d) was treated with linear geometry electrodes (pig #1 from Zmuc et al.). In sample S3, one of the electrodes was inserted into the vena cava (c) and in sample S4 into one of the major hepatic veins (d). The zones of acute changes in the liver parenchyma were identified in the microscopic images. In the work of Zmuc et al., three zones were identified: the central cavity caused by electrode insertion (Zone 1), the surrounding zone of coagulation necrosis with complete loss of liver microstructure (Zone 2), and a zone of partially damaged liver parenchyma attributed to IRE (Zone 3). In this study, we focus only on Zones 2 and 3 because Zone 1 is caused by mechanical damage by the electrodes and is always included in Zone 2. To avoid confusion regarding numbering, we changed the zoning to Zone A (Fig. 4, red arrows), which corresponds to Zones 1–2, and Zone B (Fig. 4, blue arrows), which corresponds to Zone 3 in the original article^33^. A pathologist manually outlined the two zones in microscopic images. The outlined images were imported to Adobe Illustrator CS4 where major haptic vessels, vessel walls, portal spaces and bile ducts were manually outlined as well, and electrode insertion trajectories were determined. The outlines were saved in vector format (Fig. 5a,b) and imported into COMSOL Multiphysics to construct the geometry for the numerical models.

In case of hexagonal electrodes, the sections were cut perpendicular to the electrode orientation and all 7 electrodes were visible; therefore, we were able to construct a 2D model directly from the imported vector images (Fig. 5a). Electrodes were modelled as circles with a 0.7 mm diameter. In case of linear electrodes, only one electrode of the pair was visible, and the sample was not cut perpendicular to the electrodes, therefore a 3D model was required. The vector image was imported into the work plane in the 3D model, and the vessels, bile ducts and portal spaces were modelled with geometrical primitives (cylinders and spheroids) so that the intersection of the primitives with the work plane overlapped with the outlined anatomical structures (red intersections on Fig. 5b). Electrodes were modelled as cylinders with a 1.2 mm diameter and 3 cm active length. The orientation of the first electrode was determined from the microscopic image, and the counter electrode (second electrode of the pair) was modelled completely parallel at a distance of 2 cm. Since the position of the counter electrode could not be determined from the microscopic images, we determined its position by rotating it in 5° steps around the first electrode at the circumference of 2 cm, and comparing the appearance of the computed electric field to the segmented zones (see Methods Section "Electric field distribution and threshold determination"). The angle that resulted in the highest Sørensen-Dice similarity coefficient was selected as the final model.

### Electric field distribution and threshold determination

COMSOL Multiphysics was used for computation of electric field and temperature distribution during the procedures. The computation process is described in detail in^19,34–36^. Briefly, the model consists of solving the partial differential equation for electric potential distribution in steady state form. Electrical conductivity is a non-linear function of the local electric field, and is represented by a smoothed step function, specific to each tissue modelled. To evaluate tissue heating during treatment, the modified Pennes’ bioheat equation^49^ is solved in time domain. The power dissipation density from the stationary computation is used as the heat source. The duty cycle approach is used to shorten computation times^50^. In hexagonal electrode geometry, the electric field and temperature for each electrode pair is computed separately, and the contributions from all 12 pairs are then superimposed to reproduce the final field distribution. Local conductivity increase due to electric field is independent between the pairs (each computation starts with base conductivity). The heat dissipation process is much slower than electroporation phenomenon, therefore the base conductivity for the following electrode pair computations is increased due to increased temperature. For a more detailed explanation of the modeling approach, see Supplementary materials Section "Numerical model and computation".

The computed electric field and temperature distributions were imported into MATLAB for comparison with the segmented microscopic images. The 2D model was already identical to the microscopic image of the sample (Fig. 5a), while in the 3D model, the plane corresponding to the microscopic image of the sample was extracted for evaluation (Fig. 5b). For each of the four sample models, the similarity between the computed electric field and the segmented microscopic zones was calculated using the Sørensen-Dice similarity coefficient (DSC)^51^. DSC calculates the similarity between two binary images (masks) and takes a value between 0 and 1. A similarity of 1 means that the masks match perfectly. We compared the masks of Zones A and B with masks representing isocontours of the electric field at selected thresholds (field masks). The process is shown in Fig. S2 of the supplementary materials. The masks of Zones A and B were extracted directly from the outlines of the microscopic images (Fig. S2, top row), while the field masks were obtained by applying a specific threshold to the computed electric field distribution. Thresholds ranging from 300 to 1500 V/cm were applied in steps of 10 V/cm, resulting in 121 field masks (Fig. S2, middle row). DSC was calculated between each zone mask and the 121 field masks, resulting in 121 DSC values for each zone (Fig. S2, bottom row). A higher DSC value indicates a greater similarity between the isocontour of the field obtained with the respective threshold level and the shape of the segmented zone. Therefore, the two electric field thresholds that yielded the highest DSC value were set as the thresholds for Zones A and B, respectively.

### Liver microstructure and parametric study of tissue properties

In order to evaluate, how the liver microstructures and the variability in electrical properties of these structures affect the computed electric field distribution we constructed a 2D model incorporating the hexagonal liver architecture. A hexagonal structure representing the hepatic lobules and interlobular septa was constructed using Adobe Illustrator CS4. The modelled lobule diameter was 1950 μm, CV diameter was 150 μm and septum thickness was 50 μm (Fig. 3 a). Positions of the electrodes were taken from sample S1 with parenchymal hexagonal electrodes. The liver macro- and microstructures are several magnitudes apart in size, which significantly increases the computational difficulty. Therefore, the computations were performed only in one section of the sample containing a single electrode pair (green circle in Fig. 5a). The geometry was imported into COMSOL Multiphysics where a 2D numerical model was constructed for the computation of electric field.

Three models with a varying degree of geometrical details were computed; a heterogeneous model incorporating the whole liver microstructure (hepatic lobules, septa and CVs), a semi-homogeneous model consisting of homogeneous hepatic tissue with CVs, and a fully homogeneous model consisting only of hepatic tissue. In order to eliminate error due to meshing, identical geometry (whole liver microstructure) was used in all three models; the homogeneous effect was achieved by matching the conductivity of the septa and CVs to the conductivity of the lobules.

The change in electrical conductivity due to electroporation of each modelled structure is approximated in COMSOL as a smoothed step function, with the following parameters: base electrical conductivity (σ_0_), factor of conductivity increase, center (E_C_) and size (E_W_) of the transition zone. The parameters of the electrodes and all tissues except for the septa were taken from previous works^19,27,36^, and are listed in Supplementary Table S1. The electrical conductivity of the interlobular septa is unknown, therefore, we performed a parametric study, using the heterogeneous model, where we varied all four parameters of the conductivity function. The base conductivity was defined as a fraction of base conductivity of hepatic lobules, while the factors of conductivity increase and values of E_C_ and E_W_ were taken from conductivity functions of hepatic lobules and vessel wall. All studied parameter values are listed in Supplementary Table S2.

We computed the electric field for each set of parameters of the heterogeneous model (72 combinations in total) and for the semi-homogenous and homogeneous models. Then we calculated the similarities between the computed electric fields and segmented Zones A and B of sample S1 (see Fig. 5a) and compared the thresholds determined by the different models (see Methods Section "Electric field distribution and threshold determination").

### Ethics declarations

The animal experiments were performed in the scope of our previously published animal study by Zmuc et al. (J. Zmuc et al. Sci Rep, 2019. 9:3649), which is reported in accordance with ARRIVE guidelines for reporting of research involving animals. All experiments were performed in accordance with the relevant guidelines and regulations. Regulatory approval for the study was obtained from the National Ethics Committee at The Administration of the Republic of Slovenia for Food Safety, Veterinary, and Plant Protection (U34401-1/2017/4; approval date: 17.03.2017).

## Supplementary Information

Supplementary Information.

## Data Availability

The datasets generated during and/or analysed during the current study are available from the corresponding author on a reasonable request.
