# Supplementary material for: Numerical mesoscale tissue model of electrochemotherapy in liver based on histological findings
Source: Sci Rep. 2022 Apr 20;12:6476. doi: 10.1038/s41598-022-10426-2 (PMC9021251; doi:10.1038/s41598-022-10426-2)
Supplement: Supplementary file 1 — Supplementary Information. [file 41598_2022_10426_MOESM1_ESM.pdf]

Numerical mesoscale tissue model of electrochemotherapy in liver based on histological findings

Supplementary information

Helena Cindric, Gorana Gasljevic, Ibrahim Edhemovic, Erik Brecelj, Jan Zmuc, Maja Cemazar, Alenka Seliskar, Damijan Miklavcic, Bor Kos

Numerical model and computation

The electric field distribution ***E*** in the target tissue is computed indirectly by solving the partial differential equation for electric potential *V* in steady state, governed by equations:

$$\nabla \cdot \boldsymbol{J} = 0, \tag{1}$$

$$\boldsymbol{J} = \sigma \boldsymbol{E}, \tag{2}$$

$$\boldsymbol{E} = -\nabla \mathrm{V}\,, \tag{3}$$

Where ***J*** is the current density, σ is the tissue conductivity, ***E*** is the electric field strength, and *V* is electric potential. By combining Eqs. 1-3 we get the following equation:

$$\nabla \cdot \big(\sigma(-\nabla V)\big) = 0. \tag{4}$$

The external tissue domain boundaries are set as electrically insulated. The non-linear increase of tissue electrical conductivity due to local electric field is modelled with a smoothed step function (Figure S1), which is defined by the base electrical conductivity (σ_0), factor of conductivity increase, center (E_C) and size (E_W) of transition zone. Thus, the conductivity in Eq. 4 becomes a function of the local electric field:

$$\sigma \to \sigma(|E|). \tag{5}$$

Tissue specific parameters used in our models are listed in Table S1.

In case of multiple electrode pairs (hexagonal electrodes), the electric field is calculated separately for each active pair. The conductivity increase due to electric field is independent between pairs (i.e., each computation starts with base conductivity). The final electric field distribution *in situ* is determined by superimposing the maximal electric field contribution from all electrode pairs.

The stationary model of electroporation is supplemented with the modified Pennes' bioheat transfer equation solved in the time domain:

$$\rho C_p \frac{\partial T}{\partial t} - \nabla(k\nabla T) = Q_{bio} + \sigma(|E|^2), \tag{6}$$

where *T* is temperature, ρ is tissue density, C_p is tissue thermal capacity and *k* is thermal conductivity. The bioheat source term Q_{bio} represents blood perfusion and metabolic activity; however, when electroporation occurs in tissue, perfusion decreases significantly due to vascular lock effect [1]. Heating from the electrodes is introduced via the Joule heating term. In order to reduce the computation time

we use a duty cycle approach; the Joule heating term is scaled according to the pulse duration divided by period thus effectively averaging the heating during the pulsing sequence [2].

In addition to increase due to electroporation, tissue heating also increases the electrical conductivity. The thermal dependence of electrical conductivity is modelled uniformly for all tissues with a factor of increase by 1.0 %/°C (α_T):

$$\sigma(|E|, T) = \sigma(|E|) \cdot (1 + \Delta T \cdot \alpha_T), \tag{7}$$

where ΔT is the increase from the base tissue temperature of 37°C.

In case of multiple electrode pairs (hexagonal electrodes) the base conductivity in each computation is increased due to heat generated from the previous electrode pairs. Furthermore, the vascular lock effect is modelled by decreasing the perfusion rate of tissue to 20 % of its initial value (Table S2) in the areas where the threshold for reversible electroporation is exceeded. In the large vessels the perfusion rate is not decreased.

The extent of thermal damage is determined by integrating the Arrhenius kinetics equation over the treatment time period (Eq. 8) and transformed into probability of cell death (Eq. 9) [3]:

$$\Omega(t) = \int_{t=0}^{t=t_{end}} \zeta \cdot \exp\left(\frac{-E_a}{R \cdot T(t)}\right) dt\,, \tag{8}$$

$$P = 100 \cdot \left(1 - \exp\left(-\Omega(t)\right)\right), \tag{9}$$

where ξ represents the pre-exponential frequency factor, E_a is the activation energy and R is the universal gas constant. P is the probability of cell death expressed in percentage. The threshold for thermal damage was 90 % probability of cell death as determined by Eq. 8.

Figure S1: An example of smoothed step functions, describing the dependence of tissue electrical conductivity on local electric field strength for three main tissues of the mesoscale model of liver. Function parameters are listed in Table S2. *CV – centrilobular vein*

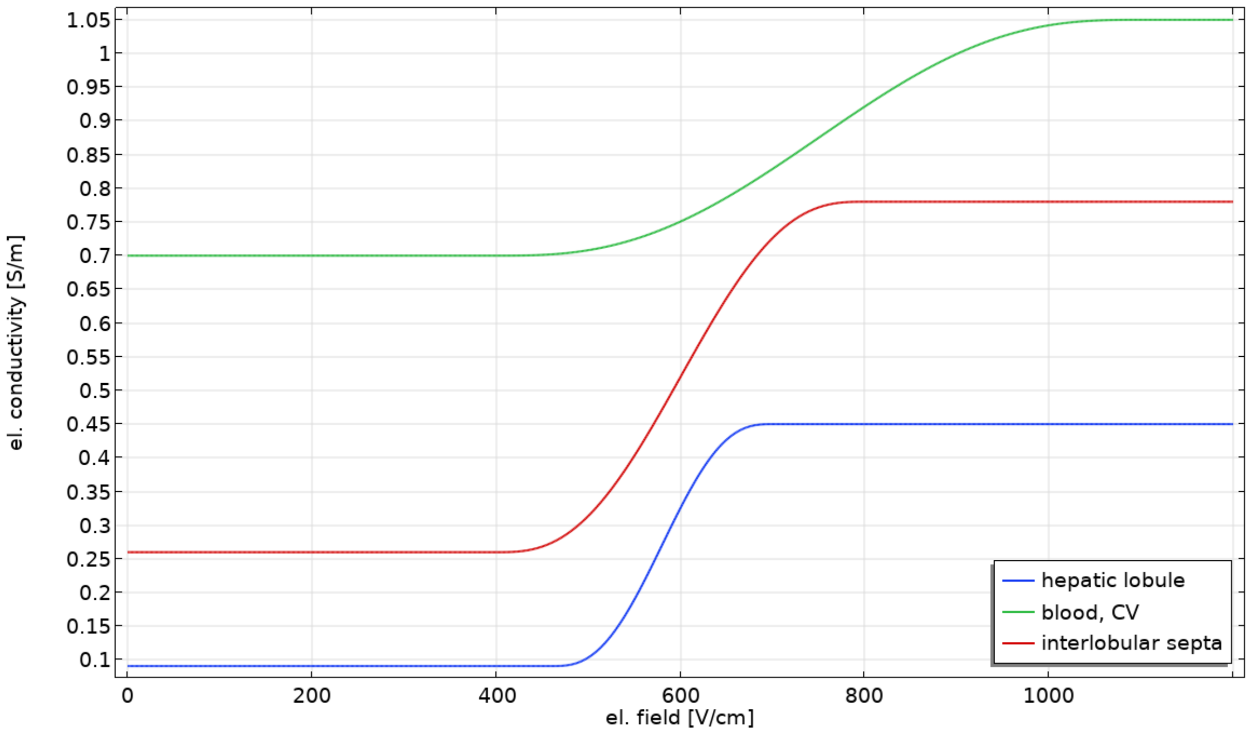

Table S1: Electrical and thermal properties of modelled tissues and electrodes.

**Tissue property**	**Hepatic lobules**	**Blood and central veins**	**Vessel walls, portal spaces, septa**[1]	**Bile**	**Electrodes**
Base electrical conductivity σ_0 [S/m]	0.091	0.70	0.26	1.47	10^6
Factor of electrical conductivity increase	4.95	1.50	3.00	/	/
Center of transition zone E_C [V/cm]	580	750	600	/	/
Size of transition zone E_W [V/cm]	240	700	400	/	/
Thermal conductivity k [W/m·K]	0.52	0.44	0.57	0.58	15
Density ρ [kg/m^3]	1079	1056	1060	928	6000
Heat capacity C_P [J/kg·K]	3540	3840	3840	4037	500
Initial perfusion ω_b [1/s]	0.017983[2]	0.2	0.2	/	/
Activation energy E_a [J/mol]	5.06×10^5	5.06×10^5	4.3×10^5	4.3×10^5	/
Frequency factor ξ *[1/s]*	2.984×10^{80}	2.984×10^{80}	5.6×10^{63}	5.6×10^{63}	/
References	[4]	[4–6]	[5, 6]	IT'IS database	

[1] Interlobular septa properties were varied in the parametric study (Table S2). An example of the conductivity function is shown on Figure S1.

[2] Where the local electric field strength exceeds the threshold for electroporation (E_0) perfusion decreases to 20 % its' initial value.

Determining the electric field thresholds for Zones A and B

Figure S2: Top row: outlines of Zones A and B from histological images are converted into two binary masks. Middle row: 121 thresholds (300-1500 V/cm in steps of 10 V/cm) are applied to the computed electric field distribution, resulting in 121 binary field masks. Bottom row: the Sørensen-Dice similarity coefficient (DSC) is calculated between the mask of each zone and all 121 field masks, resulting in 121 DSC values for each zone. The maximum DSC values for both zones are marked.

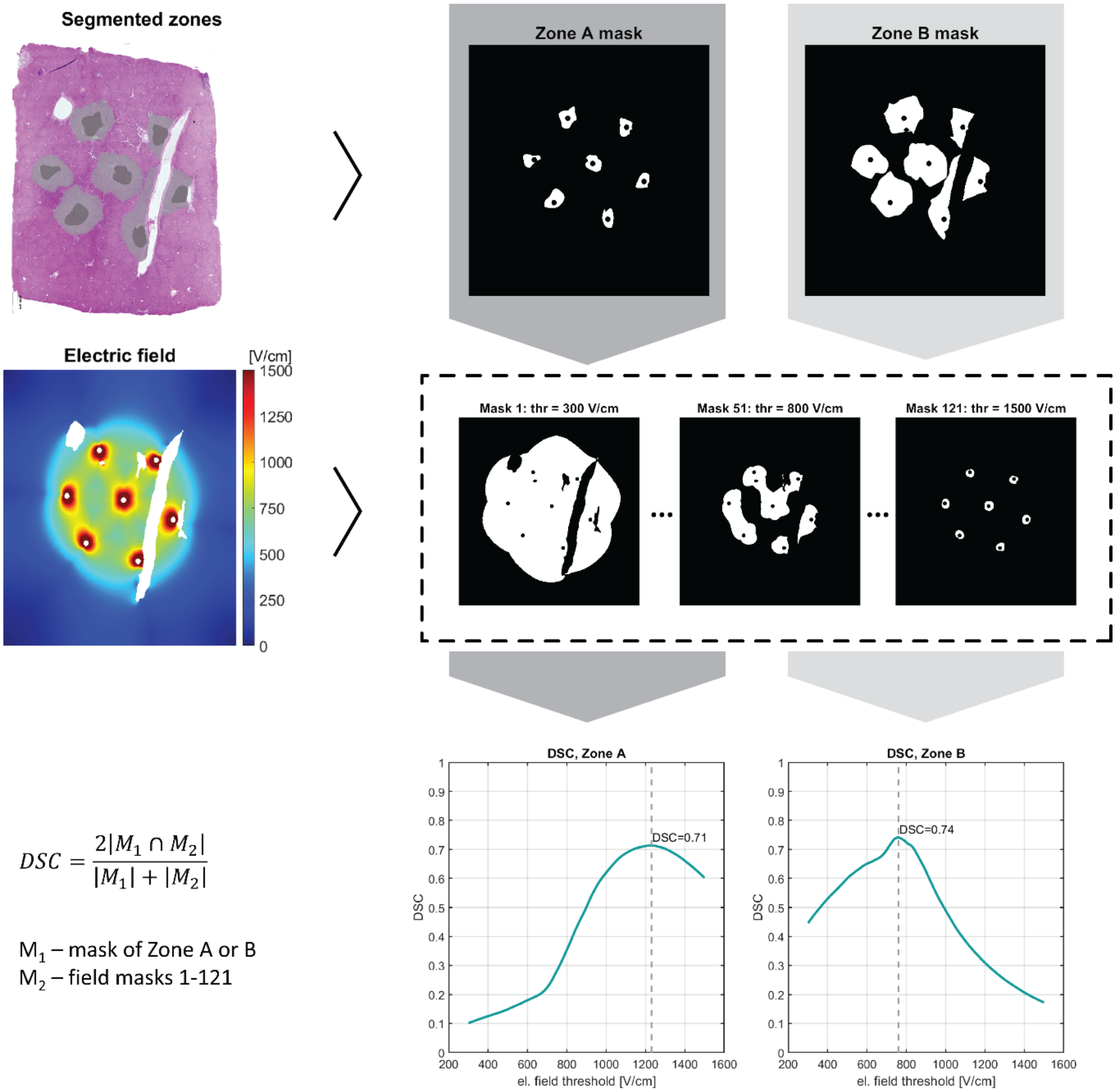

$$DSC = \frac{2|M_1 \cap M_2|}{|M_1| + |M_2|}$$

M_1 – mask of Zone A or B
M_2 – field masks 1-121

Parametric study details

We varied parameters defining the smoothed step function of the interlobular septa domains. Parameters of all other tissues were fixed and are shown in Table S1.

Table S2: Parameter values of the parametric study of conductivity function of the interlobular septa

Parameter	Parameter value
Base gap conductivity $\sigma_{0,SEPTA}$	(0.1, 0.5, 0.75, 1, 2, 2.86) · $\sigma_{0,LOBULE}$
Factor of conductivity increase due to EP	3, 3.5, 5
Center of transition zone	580 V/cm, 600 V/cm
Size of transition zone	240 V/cm, 400 V/cm

Thermal simulation results

Figure S3: Map of computed temperature distribution in tissue after electrochemotherapy treatment.

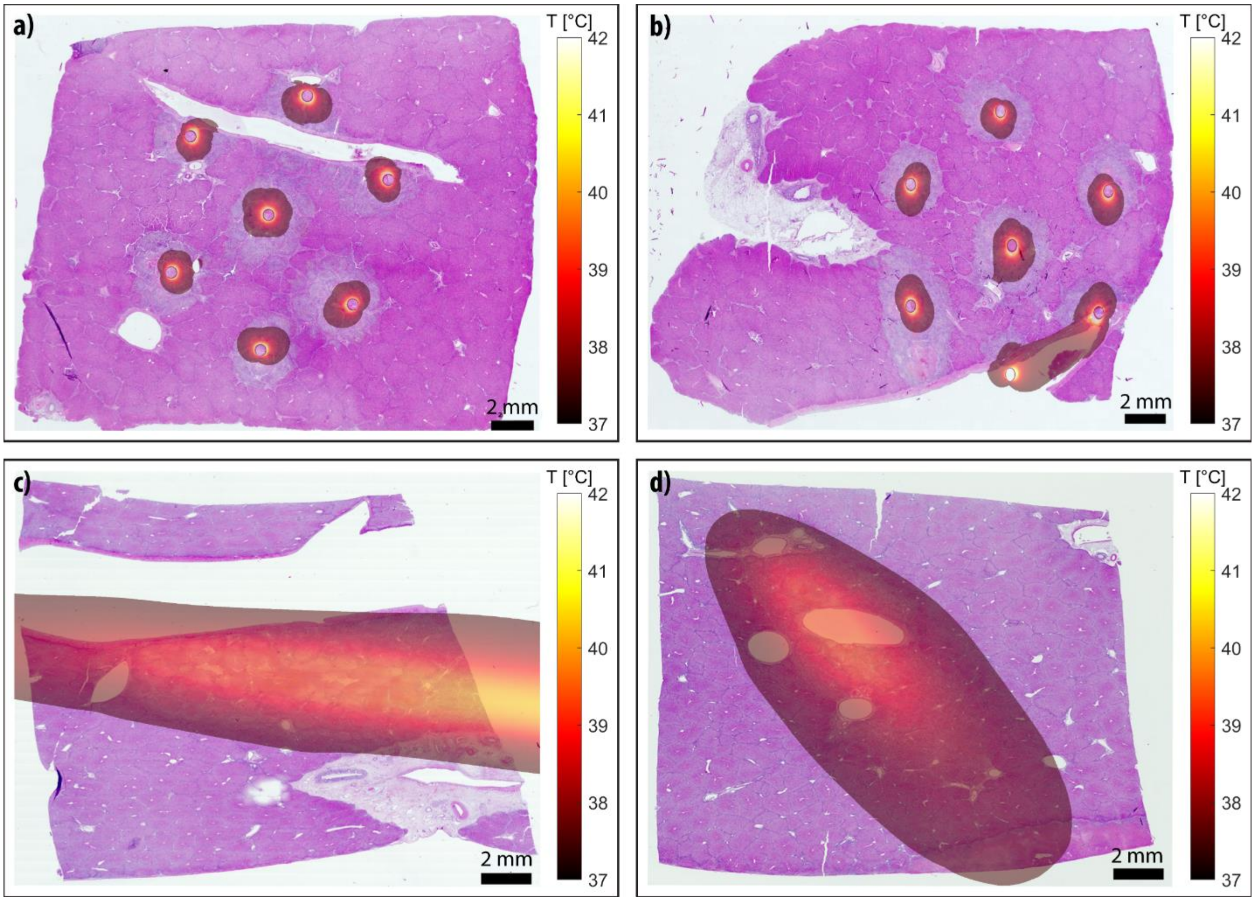

References

1. Jarm T, Cemazar M, Miklavcic D, Sersa G (2010) Antivascular effects of electrochemotherapy: implications in treatment of bleeding metastases. Expert Rev Anticancer Ther 10:729–746. https://doi.org/10.1586/era.10.43

2. Neal RE, Garcia PA, Robertson JL, Davalos RV (2012) Experimental characterization and numerical modeling of tissue electrical conductivity during pulsed electric fields for irreversible electroporation treatment planning. IEEE Trans Biomed Eng 59:1076–1085. https://doi.org/10.1109/TBME.2012.2182994

3. Garcia PA, Davalos RV, Miklavcic D (2014) A numerical investigation of the electric and thermal cell kill distributions in electroporation-based therapies in tissue. PLoS ONE 9:e103083. https://doi.org/10.1371/journal.pone.0103083

4. Kos B, Voigt P, Miklavcic D, Moche M (2015) Careful treatment planning enables safe ablation of liver tumors adjacent to major blood vessels by percutaneous irreversible electroporation (IRE). Radiol Oncol 49:234–241. https://doi.org/10.1515/raon-2015-0031

5. Duck FA (2012) Physical Properties of Tissue: A Comprehensive Reference Book. Institute of Physics and Engineering in Medicine

6. Marčan M, Kos B, Miklavčič D (2015) Effect of Blood Vessel Segmentation on the Outcome of Electroporation-Based Treatments of Liver Tumors. PLOS ONE 10:e0125591. https://doi.org/10.1371/journal.pone.0125591
